# Non-alcoholic fatty liver disease (Nafld) in obese children- effect of refined carbohydrates in diet

**DOI:** 10.1186/s12887-016-0726-3

**Published:** 2016-11-15

**Authors:** Deise Rosa Félix, Fabiola Costenaro, Catarina Bertaso Andreatta Gottschall, Gabriela Perdomo Coral

**Affiliations:** 1 Postgraduate Program in Medicine: Hepatology, Universidade Federal de Ciências da Saúde de Porto Alegre, 91720-030 Porto Alegre, RS Brazil; 2 Hospital da Criança Santo Antônio de Porto Alegre, Porto Alegre, Brazil; 3 Universidade Federal de Ciências da Saúde de Porto Alegre, Porto Alegre, Brazil

**Keywords:** Obesity, Nonalcoholic fatty liver disease, NAFLD, Children

## Abstract

**Background:**

The incidence of childhood obesity has increased progressively and, associated with this, nonalcoholic fatty liver disease (NAFLD) has often been diagnosed in this age group. To determine the risk factors associated with NAFLD in obese children, with special emphasis on diet.

**Methods:**

A prospective cross-sectional study was conducted with obese children referred to the endocrinology outpatient clinic. Questions about dietary habits and physical activity were applied. In addition, two 24 h food recalls were collected. Anthropometric measurements, biochemical tests and abdominal ultrasound were obtained. The study was approved by the institutional review board of Irmandade Santa Casa de Misericórdia de Porto Alegre Hospital (ISCMPA). A 5% statistical significance level was considered statistically significant.

**Results:**

Of 55 patients initially allocated, 39 were evaluated and 8 (20.5%) had a diagnosis of NAFLD, which was more prevalent among boys (87.5%). Logistic regression analysis showed that the predictive factors independently associated with the presence of NAFLD were male gender (OR: 1.62; 95% CI: 1.08– 2.44; *p* = 0.038); high amount of refined carbohydrates in the diet (OR: 2.17; 95% CI: 1.05 – 6.82; *p* = 0.038) and absence of routine physical activity (OR: 3.35; 95% CI:1.97 – 0.006; *p* = 0.006).

**Conclusions:**

The prevalence of NAFLD in obese children in our series was high. Furthermore, the high amount of refined carbohydrates in the diet, male gender and sedentary lifestyle were significant risk factors for its occurrence.

## Background

Obesity in childhood and adolescence is following the widespread epidemic of obesity in adulthood. It is estimated that at least 155 million children worldwide are overweight or obese [1]. Studies show that childhood obesity is an important predictor of obesity and several comorbidities in adulthood. Furthermore, pediatric obesity is associated with nonalcoholic fatty liver disease (NAFLD), a leading cause of liver disease [2, 3]. The term NAFLD refers to a spectrum of liver disease, ranging from simple steatosis to nonalcoholic steatohepatitis (NASH), that includes hepatic inflammation and can result in various degrees of fibrosis and cirrhosis, even in children [4].

Diet composition is an environmental factor that might influence NAFLD occurrence and severity, but only few studies correlate diet and NAFLD in children [5, 6]. A recent study that included 149 children and adolescents with NAFLD did not find an association between diet and severity of NAFLD, but this issue requires further studies [7].

To our knowledge there are only three studies of NAFLD in the pediatric population in Brazil [8–10]. The high prevalence of obesity in this population and potential risk of diseases in adulthood, like diabetes mellitus and cirrhosis, highlights the need of studies to assess the risk factors for NAFLD in this population. The knowledge of risk factors can also guide future interventions.

## Methods

This is a prospective, cross-sectional study assessing patients referred to the endocrinology outpatient clinic of a pediatric hospital at Porto Alegre, Brazil, in the period between June 2010 and April 2013.

Patients with age of three to fourteen and obesity were invited to participate, receiving a detailed explanation of the study and were included if their parents or relatives signed an informed consent form.

They were previously screened for the presence of HBsAg antigens, HCV antibodies, antimitochondrial antibodies, antinuclear antibodies. Wilson disease, Alfa 1 antitrypsin deficiency, hemochromatosis and other genetic liver diseases were excluded. Patients with another liver disease besides NAFLD were not included.

Initially, patients or their guardians answered questions about food history (duration of exclusive breastfeeding, amount of fruits, vegetables, candies and water consumption). Regarding to physical activity, the child’s routine activities during the previous day and the practice of any physical activity were evaluated. Children that didn’t do any kind of physical activity were defined as sedentary. Dietary habits were recorded through questions on the daily intake of lipids, carbohydrates, proteins, fruits, vegetables, sweets and water, and by two 24 h food recalls (one during the week and another at the weekend) to determine the interindividual variation in nutrient intake. Portions were estimated by using a photo album of utensils and food, prepared especially for the research.

Dietary data were obtained using the Diet Win Nutrition Support Program and food composition tables [11], including the Brazilian table of food composition [12], in addition to information obtained from labels and contact with food industries. The average values of food consumption, obtained with the two 24 h recalls and using the reference values established for this age group, were used for the analyses [13, 14].

Weight was measured with patients barefoot and in light clothing, such as shorts for boys and shorts and T-shirts for girls, according to accepted international procedures, and using a Toledo® electronic scale with a capacity of 120 kg and precision of 100 g. Height was measured using a Seca® stadiometer with a 0.01 cm accuracy, set in a smooth wall, with the patient in vertical position, with feet parallel and with the heels, shoulders and buttocks touching the wall. Body mass index (BMI) was calculated as weight divided by height squared. The presence of obesity was diagnosed with BMI levels ≥ to the 95th percentile, considering the percentile curves recommended by the World Health Organization, 2007 [15]. Hepatic steatosis was diagnosed by upper abdominal ultrasound when there was diffuse increased echogenicity of the liver [16]. The diagnosis of NAFLD was established in the presence of hepatic steatosis by ultrasound regardless of the aminotransferase values; the radiologist was blinded to clinical data.

For quality control, the data were entered twice, with confirmation by telephone calls in 20% of the sample. The study was approved by the institutional review board of Irmandade Santa Casa de Misericórdia de Porto Alegre Hospital (ISCMPA).

Statistical analysis of the data was performed using the software SPSS (Statistical Package for the Social Sciences) version 17.0. The quantitative variables were described as mean and standard deviation (symmetric distribution) or median and interquartile range (asymmetric distribution). Categorical variables were described as absolute and relative frequencies. Means were compared with the Student’s t test, and in case of asymmetry the MannWhitney test was used. The Fisher exact test was used to assess the association between categorical variables. Binary logistic regression, backward model, was used for estimation of the odds ratio (OR) of the factors associated with the diagnosis of NAFLD. A 5% statistical significance level was considered statistically significant.

## Results

A total of 55 obese patients were initially investigated, but sixteen were lost to follow up and were excluded from the study, therefore 39 were included. Eight of the 39 patients (20.5%) were diagnosed with NAFLD.

The average age was 8.8 ± 2.5 years, and median age was 9 years (3–14 years). Seventeen patients (43.6%) were male and 22 (56.4%) female. All patients were obese.

Daily dietary patterns are presented in Table 1. Thirty patients (81.1%) were exclusively breastfed for less than six months, 31 (79.5%) consumed sweets daily, and 16 (41.0) and 19 (48.7%) did not ingest any kind of fruit or vegetables, respectively. In two patients the duration of breastfeeding could not be determined, since in one case the child was adopted and in the other, the grandmother was the guardian and could not provide the information. The average daily consumption of water was 943.2 ± 646.1 ml. Only 11 patients (28.2%) reported having five or more daily meals. Twenty-nine of patients (74.4%) didn’t have an adequate ingestion of fibers.Almost fifty per cent of children consumed an appropriate amount of lipids and the amount of protein ingestion was adequate in 94.9%. Finally, inadequate consumption of refined carbohydrates was present only in 15.4%, but in bivariate analysis there was a trend for association between high amount of refined carbohydrates and the diagnosis of NAFLD (*p* = 0.08). Vitamins C and E have been reported as adequate in 74.4 and 69.2% respectively and daily consumption of calories was inadequate in all cases, with an average of 2,831 ± 725.5 kcal and a median of 2,726Kcal. There were no statistically significant differences between these profiles of food intake and dietary habits between patients with NAFLD.

**Table 1:** Association between diet pattern and the presence of NAFLD

Variables	Total (*n* = 39) *	NAFLD**		*p*(value)
		Present (*n* = 8)	Absent (*n* = 31)	
Breastfeeding				
Yes	7 (18.9%)	2 (25.0%)	5 (17.2%)	0.631 ¶
No	30 (81.1%)	6 (75.0%)	24 (82.8%)	
Sweets				
Yes	31 (79.5%)	6 (75.0%)	25 (80.6%)	0.658 ¶
No	8 (20.5%)	2 (25.0%)	6 (19.4%)	
Fruits				
Yes	23 (59.0%)	4 (50.0%)	19 (61.3%)	0.694 ¶
No	16 (41.0%)	4 (50.0%)	12 (38.7%)	
Vegetables				
Yes	20 (51.3%)	5 (62.5%)	15 (48.4%)	0.695 ¶
No	19 (48.7%)	3 (37.5%)	16 (51.6%)	
Water Consumption				
Mean ± standard deviation	943.2 ± 646.1	1300.0 ± 1050.3	860.0 ± 502.1	0.505
Median (Range)	800 (200–3000)	1000 (200–3000)	700 (200–2000)	
More Than 5 Meals				
Yes	11 (28.2%)	3 (37.5%)	8 (25.8%)	0.663 ¶
No	28 (71.8%)	5 (62.5%)	23 (74.2%)	
Appropriate Lip				
Yes	18 (46.2%)	3 (37.5%)	15 (48.4%)	0.702 ¶
No	21 (53.8%)	5 (62.5%)	16 (51.6%)	
Appropriate Vit C				
Yes	29 (74.4%)	5 (62.5%)	24(77.4%)	0.399 ¶
No	10 (25.6%)	3 (37.5%)	7 (22.6%)	
Appropriate Vit E				
Yes	27 (69.2%)	7 (87.5%)	20 (64.5%)	0.394 ¶
No	12 (30.8%)	1 (12.5%)	11 (35.5%)	
Total energy value				
Mean ± standard deviation	2831.0 ± 725.5	2838.5 ± 658.9	2829.0 ± 751.93	1.000
Median (Range)	2726 (1713–4486)	2763.5 (1953–3798)	2726 (1713–4486)	
% Refined Ch in diet				
Normal	33 (84.6%)	5 (62.5%)	28 (90.3%)	0.088 ¶
Altered	6 (15.4%)	3 (37,5%)	3 (9.7%)	
% Refined PTN in diet				
Normal	37 (94,9%)	7 (87,5%)	30 (96,8%)	0.372 ¶
Altered	2 (5,1%)	1 (12,5%)	1 (3,2%)	

*Values presented as *n* (%) with percentages obtained from total sample; **Values presented as *n* (%) with percentages from the total of each NAFLD category; ¶ Fisher’s exact test for independent groups assuming equal variances

Table 2 demonstrated the demographic, clinical and dietary characteristics associated with NAFLD in bivariate analysis. Seven of eight patients with NAFLD (87.5%) were boys. The mean and median age of patients was 8.8 and 9 respectively; there was an association between older children and the diagnosis of NAFLD. Patients with NAFLD were even more obese in comparison with those without the disease. The mean weight and standard deviation was 72.2 ± 18.6 kg and 49.9 ± 15.7 kg, respectively) *p* = 0.001). Patients without regular physical activity had NAFLD more frequently; 87.5% [7] of children with NAFLD performed no physical activity. In one patient this information could not be assessed, since the guardian could not provide reliable information regarding physical activity outside the school environment.

**Table 2:** Characteristics associated with NAFLD in bivariate analysis

Characteristics	Total (*n* = 39) *	NAFLD**		*p*(valor)
		Present (*n* = 8)	Absent (*n* = 31)	
Gender				
Female	22 (56.4%)	1 (12.5%)	21 (67.7%)	0.013 ¶
Male	17 (43.6%)	7 (87.5%)	10 (32.3%)	
Age				0.023 ^a^
Mean	8.8	10.6	8.3	
Physical activity				
Yes	14 (36.8%)	1 (12.5%)	13 (43.3%)	0.108 ¶
No	24 (63.2%)	7 (87.5%)	17 (56.7%)	
% Refined Ch in diet				
Normal	33 (84.6%)	5 (62.5%)	28 (90.3%)	0.088 ¶
Altered	6 (15.4%)	3 (37,5%)	3 (9.7%)	

*Values presented as *n* (%) with percentages from total sample; **Values presented as *n* (%) with percentages from the total of each NAFLD category; ¶ Fisher’s exact test; ^a^Student’s t test for independent groups assuming equal variances; *CH* carbohydrates

After logistic regression, the predictive factors independently associated with the presence of NAFLD were male gender (OR: 1.62; 95% CI: 1.08 – 2.44; *p* = 0.038); sedentary lifestyle (OR: 3.35; 95% CI:1.97 – 0.006; *p* = 0.006); and inadequate amount of refined carbohydrates in diet (OR: 2.17; 95% CI: 1.05 – 6.82; *p* = 0.038) (Table 3).

**Table 3:** Risk estimation for NAFLD with the binary logistic regression model, according to variables listed as predictors – backward conditional model

NAFLD predictive factors (1)	Odd ratios		
	OR	95% CI	*P*
Gender			
Male	1,62	1,08–2,44	0,038
Physical activity			
No	3,35	1,97–11,76	0,006
% Refined CH in diet			
Altered	2,17	1,05–6,82	0,038

(1) Pseudo-*R* ^*2*^ = 0.431; “-2 Likelihood = 88.254; Hosmer and Lemeshow (*p* = 0.688); Pearson’s Chi-square ((χ^2^ = 28.859; *p* < 0.001). Adjusted to age group (22) CH = carbohydrates

## Discussion

The prevalence of NAFLD in the pediatric age group is estimated to be 3 to 10% of the world’s population, but it can reach 80% among obese children [17]. It is estimated that this percentage is influenced by the characteristics of the population, especially life habits, as well as the methods used to diagnose it. However, despite the diversity of diagnostic criteria used in population based studies, obesity is the main risk factor for NAFLD in children [18]. For these reason, we evaluated only obese children to establish the risk factors related to NAFLD in these subgroup. It is well-known that dietary habits are relevant in NAFLD. A study analyzing 43 adolescents showed a higher consumption of carbohydrates, protein and cholesterol among NAFLD patients. On the other hand, there was no significant difference in the consumption of lipids between the two groups, but there was a positive association between visceral obesity and consumption of lipids in patients with nonalcoholic steatohepatitis [5].

The present study showed no association between total energy value and the presence of NAFLD, although the average consumption was close to three thousand calories a day; it should be stressed that all children in the sample were obese. Other studies have also shown a lack of association between caloric intake and the presence of NAFLD [5, 6].

The increased consumption of refined carbohydrates indicated a 2.17 fold risk for the occurrence of NAFLD in this study. This finding was not observed in some studies [5, 6]. However, similar to our results, Papandreou et al. reported that the total carbohydrate intake was significantly higher in patients with NAFLD (288.8 ± 70.6 g) compared to individuals without NAFLD (244.5 ± 67.5 g) (*P* 0.001) [19].

Regarding the consumption of fruits, Hattar et al. evaluated 57 individuals aged between 8 to 16 years. Patients with NAFLD consumed less fruit when compared to patients with normal weight and obese; only 25% of NAFLD patients consumed one or more fruits a day, compared with 45% of obese and 64.7% of those with normal weight [20]. In the present series, we were not able to demonstrate any associations of insufficient intake of fruits or vegetables and presence of NAFLD. Nobili et al., demonstrated a protective effect of breastfeeding in NAFLD. They studied 191 children and observed a lower risk of NASH and fibrosis in breastfed participants, with a decreased risk for each further month of breastfeeding [21]. In the present study, we did not find a correlation between breastfeeding and NAFLD, however approximately 80% of children were not exclusively breastfed until the sixth month.

Papandreou et al. demonstrate a strong association among BMI, waist circumference and NAFLD studying 82 patients (8–15 years). In addition, levels of BMI were higher in children with severe disease (37.2 ± 6.2 kg/m2 and 102.9 ± 14.0 cm) compared to mild NAFLD (26.6 ± 3.3 kg/m2 and 86.1 ± 9.9 cm, respectively) [19]. In the present study we did not demonstrate an association between BMI and NAFLD at logistic regression, but all patients included were already obese.

It has been shown that physical activity has a protective effect against NAFLD. In the present study, children who were not physically active had a 3.35-fold risk of NAFLD when compared to those who practiced physical exercise. A recent study evaluating three groups (eutrophic, obese and children with NAFLD) showed that the average score of physical activity was lower in the NAFLD group, but the average score for sedentary lifestyle was not significantly different among the groups [20].

This study has some limitations, such as the number of patients evaluated, the loss of patients to follow up and a possible selection bias (patients referred by an endocrinology clinic). On the other hand, this study presents a comprehensive investigation of possible factors associated with childhood obesity and NAFLD, such as dietary habits and routine physical activities and therefore it supports the importance of a simple change in lifestyle.

## Conclusions

In conclusion, we demonstrated that high amount of refined carbohydrates and sedentary lifestyle was correlated with NAFLD in this subgroup of pediatric obese patients. These results emphasize the importance of primary care and screening of these patients, particularly in male children.

## Abbreviations

BMI: Body mass index
ISCMPA: Irmandade Santa Casa de Misericórdia de Porto Alegre Hospital
NAFLD: Nonalcoholic fatty liver disease
NASH: Nonalcoholic steatohepatitis
OR: Odds ratio
SPSS: Statistical package for the social sciences
